# MAP3K19 Affects TWEAK-Induced Response in Cultured Bronchial Epithelial Cells and Regulates Allergic Airway Inflammation in an Asthma Murine Model

**DOI:** 10.3390/cimb45110559

**Published:** 2023-11-08

**Authors:** Yuuki Sandhu, Norihiro Harada, Sonoko Harada, Takayasu Nishimaki, Hitoshi Sasano, Yuki Tanabe, Tomohito Takeshige, Kei Matsuno, Ayako Ishimori, Yoko Katsura, Jun Ito, Hisaya Akiba, Kazuhisa Takahashi

**Affiliations:** 1 Department of Respiratory Medicine, Juntendo University Faculty of Medicine and Graduate School of Medicine, Tokyo 113-8421, Japan; ysando@juntendo.ac.jp (Y.S.); snharada@juntendo.ac.jp (S.H.); t-nishimaki@juntendo.ac.jp (T.N.); h-sasano@juntendo.ac.jp (H.S.); yutanabe@juntendo.ac.jp (Y.T.); takeshig@juntendo.ac.jp (T.T.); kmatsuno@juntendo.ac.jp (K.M.); aisimori@juntendo.ac.jp (A.I.); ykatura@juntendo.ac.jp (Y.K.); moisture@juntendo.ac.jp (J.I.); kztakaha@juntendo.ac.jp (K.T.); 2 Research Institute for Diseases of Old Ages, Juntendo University Faculty of Medicine and Graduate School of Medicine, Tokyo 113-8421, Japan; 3 Atopy (Allergy) Research Center, Juntendo University Faculty of Medicine and Graduate School of Medicine, Tokyo 113-8421, Japan; 4 Department of Immunology, Juntendo University Graduate School of Medicine, Tokyo 113-8421, Japan; hisaya@juntendo.ac.jp

**Keywords:** asthma, MAP kinase kinase kinases, TWEAK, transforming growth factors-β, chemokine CCL5

## Abstract

The mitogen-activated protein kinase (MAPK) signaling pathway is involved in the epithelial–mesenchymal transition (EMT) and asthma; however, the role of mitogen-activated protein kinase kinase kinase 19 (MAP3K19) remains uncertain. Therefore, we investigated the involvement of MAP3K19 in in vitro EMT and ovalbumin (OVA)-induced asthma murine models. The involvement of MAP3K19 in the EMT and the production of cytokines and chemokines were analyzed using a cultured bronchial epithelial cell line, BEAS-2B, in which MAP3K19 was knocked down using small interfering RNA. We also evaluated the involvement of MAP3K19 in the OVA-induced asthma murine model using Map3k19-deficient (MAP3K19^−/−^) mice. Transforming growth factor beta 1 (TGF-β1) and tumor necrosis factor-like weak inducer of apoptosis (TWEAK) induced the MAP3K19 messenger RNA (mRNA) expression in the BEAS-2B cells. The knockdown of MAP3K19 enhanced the reduction in E-cadherin mRNA and the production of regulated upon activation normal T cell express sequence (RANTES) via stimulation with TWEAK alone or with the combination of TGF-β1 and TWEAK. Furthermore, the expression of MAP3K19 mRNA was upregulated in both the lungs and tracheas of the mice in the OVA-induced asthma murine model. The MAP3K19^−/−^ mice exhibited worsened eosinophilic inflammation and an increased production of RANTES in the airway epithelium compared with the wild-type mice. These findings indicate that MAP3K19 suppressed the TWEAK-stimulated airway epithelial response, including adhesion factor attenuation and RANTES production, and suppressed allergic airway inflammation in an asthma mouse model, suggesting that MAP3K19 regulates allergic airway inflammation in patients with asthma.

## 1. Introduction

Mitogen-activated protein (MAP) kinases control various cellular activities throughout the body [1]. The signaling pathways of MAP kinases involve a cascade of protein kinases comprising three components. The first component is the serine–threonine protein kinase known as the MAP3-kinase, which phosphorylates and activates a dual-specificity protein kinase called the MAP2-kinase. This activated MAP2-kinase then phosphorylates and activates the MAP kinase. Among these components, MAP3-kinase 19 (MAP3K19), also known as YSK4 and RCK (regulated in the chronic obstructive pulmonary disease (COPD) kinase), is a newly discovered MAP kinase kinase kinase present in humans and mice. It is highly expressed in the lung and trachea, particularly in the bronchial epithelial cells, type II pneumocytes, and pulmonary macrophages [2,3]. Boehme SA et al. have demonstrated that MAP3K19 was also overexpressed in the alveolar macrophages of patients with idiopathic pulmonary fibrosis and in the alveolar macrophages, bronchial epithelia, and inflammatory cells of patients with COPD [3,4]. These studies demonstrated that MAP3K19 inhibition—using small interfering RNA (siRNA) and inhibitors directed at this kinase—inhibited the nuclear translocation of activated phospho-Smad2/3, following the transforming growth factor (TGF)-β stimulation of cells in the lung tissue of patients with interstitial pneumonia. It attenuated a bleomycin-induced pulmonary fibrosis murine model [3]. A few other studies have further shown that MAP3K19 inhibition attenuated the profibrotic activity of these cells in vitro and in an adoptive transfer severe combined immunodeficiency (SCID) model of pulmonary fibrosis [5]. Moreover, Boehme SA et al. have also shown that the exogenous expression of MAP3K19 in cells transiently transfected with a MAP3K19 expression plasmid upregulated nuclear factor kappa-light-chain-enhancer of activated B cells (NF-κB)-driven transcription [4]. Researchers have observed a reduction in cigarette smoke-induced inflammation in murine models by inhibiting the activity of MAP3K19 using siRNA or inhibitors. This reduction includes a decrease in pulmonary neutrophilia and the mitigation of emphysematous changes in the airway parenchyma [4]. Furthermore, the MAP kinase (MAPK) signaling pathway is involved in the epithelial–mesenchymal transition (EMT), and MAP3K19 inhibition attenuated the TGF-β-induced upregulation of mesenchymal markers, including N-cadherin [3,6]. However, the MAP3K19 kinase still has the lowest number of publications available in the MAP3K family [7], its function has not been determined, and its role in airway inflammation, including asthma, remains unclear.

Asthma usually presents with chronic allergic airway inflammation that involves epithelial injury, repair, and remodeling [8,9]. Following an injury, epithelial repair involves a complex series of events that immediately initiate and ultimately result in the organized replenishment of epithelial cells. The repetitive damage caused by airway inflammation disrupts the epithelium. It hampers its regenerative abilities, leading to remodeling characterized by basement membrane thickening, mucous cell metaplasia, and the proliferation of smooth muscle cells and lung fibroblasts, among other changes. The exact mechanisms of chronic allergic airway inflammatory disorders remain unclear; however, it is believed that the EMT is involved in the progression from bronchial epithelial repair to remodeling in individuals with asthma [10,11]. The EMT is vital in embryonic development, tissue remodeling, and wound repair [12]. As part of the EMT process, epithelial cells undergo a downregulation of E-cadherin, an epithelial marker, while concurrently upregulating the expression of N-cadherin, a mesenchymal marker. This transition enables enhanced motility in the cells [13,14]. Transforming growth factor beta 1 (TGF-β1), a multifunctional cytokine belonging to the TGF superfamily, is upregulated in airway remodeling tissues [10,11,15]. Tumor necrosis factor-like weak inducer of apoptosis (TWEAK) and its receptor fibroblast growth factor-inducible 14 (Fn14) have emerged in the tumor necrosis factor (TNF) superfamily, promoting NF-κB activation, and the TWEAK/Fn14 interaction contributes to pro-inflammatory effects and the EMT process in the bronchial epithelium [6,16,17,18]. TWEAK is produced in various cell types, including monocytes, macrophages, dendritic cells, T cells, natural killer cells, and airway smooth muscle cells [16,19], and it is significantly elevated in patients with inflammatory diseases and cancer [20,21,22,23]. Our previous reports showed that TWEAK induces adhesion factor attenuation and enhances the TGF-β1-induced EMT in bronchial epithelial cells [6]. Moreover, TWEAK and co-stimulation with TWEAK and TGF-β1 induce thymic stromal lymphopoietin (TSLP) and regulated upon activation normal T cell express sequence (RANTES) production during the EMT in bronchial epithelial cells [18]. TSLP mediates type 2 inflammation, including T helper (Th2) cells and group 2 innate lymphoid cell-promoting activity [24,25]. RANTES is a known ligand for CC chemokine receptor (CCR)1 and CCR3 expressed on the cell surfaces of eosinophils and is involved in the chemoattractant effect and activity on eosinophils [26,27]. Furthermore, it has been reported that the TWEAK levels were higher in airway smooth muscle cells from patients with asthma and in sputa from children with asthma, compared with those in non-asthmatics [19,28]. In children with asthma, the sputum TWEAK expression significantly correlated with the asthma severity, control, and airway limitation, and distinctively in children with non-eosinophilic inflammation [28].

NF-κB-inducing kinase/MAP3K14 is known as a kinase involved in NF-κB activation [29]; however, MAP3K19’s role in inflammation is yet to be determined. We hypothesized that MAP3K19 is involved in downstream TWEAK stimulation and airway inflammation in patients with asthma because TWEAK can activate NF-κB and enhance the TGF-β1-induced EMT [6,17,18]. This study explored the involvement of MAP3K19 in the EMT and the production of TSLP and RANTES using the cultured bronchial epithelial cell line BEAS-2B, in which MAP3K19 was knocked down using siRNA. We also investigated whether MAP3K19 knockdown attenuated the airway inflammation in Map3k19-deficient (MAP3K19^−/−^) mice using the OVA-induced asthma murine model.

## 2. Materials and Methods

### 2.1. Reagents

Recombinant soluble human TWEAK and TGF-β1 were purchased from Peprotech (Rocky Hill, NJ, USA). Anti-CD170 (Siglec F) monoclonal antibodies (mAbs) (1RNM44N) and anti-RANTES mAbs (25H14L17) were obtained from Invitrogen (Carlsbad, CA, USA); anti-E-cadherin mAbs (610181) were obtained from BD Biosciences (San Jose, CA, USA); anti-TSLP mAbs (#258136) and anti-TSLP and anti-TWEAK polyclonal antibodies were obtained from R&D systems (Minneapolis, MN, USA); anti-β-actin (2D4H5) and anti-GAPDH mAbs (5A12) were obtained from FUJIFILM (Tokyo, Japan). Anti-CD11c mAbs (D1V9Y), anti-extracellular signal-regulated kinase (ERK), anti-phospho-ERK (Thr202/Tyr204), anti-p38 MAPK, anti-phospho-p38 MAPK (Thr180/Tyr182) polyclonal antibodies, and anti-Jun N-terminal kinase (JNK) and anti-phospho-JNK (Thr183/Tyr185) mAbs were obtained from Cell Signaling Technology (Beverly, MA, USA). Anti-CD3 mAbs (ab16669) and anti-MAP3K19 polyclonal antibodies (A14340) were obtained from Abcam (Cambridge, MA, USA). Bronchial epithelial growth medium (BEGM) was purchased from Cambrex (East Rutherford, NJ, USA).

### 2.2. Cell Culture

The BEAS-2B cell line, a normal human bronchial epithelial cell line transformed by SV40, was obtained from ATCC (Rockville, MD, USA). The complete BEGM was used to culture the BEAS-2B. This medium consisted of bronchial epithelial basal medium (BEBM) supplemented with insulin (5 µg/mL), hydrocortisone (0.5 µg/mL), transferrin (10 µg/mL), triiodothyronine (6.5 ng/mL), epinephrine (0.5 µg/mL), human epidermal growth factor (0.5 ng/mL), retinoic acid (0.1 ng/mL), gentamicin (50 µg/mL), and bovine pituitary extract (52 µg/mL). The culture medium was changed to fresh BEBM without growth factor and serum, and recombinant soluble human TGF-β1 (10 ng/mL) or TWEAK (100 ng/mL) was added, as described in the Results section.

### 2.3. Mice

Female C57BL/6 mice were purchased from CLEA Japan (Tokyo, Japan). MAP3K19^−/−^ mice were provided by Nippon Boehringer Ingelheim Co., Ltd. (Tokyo, Japan). Polymerase chain reaction (PCR) genotyping was performed using mouse tail genomic DNAs to verify MAP3K19^−/−^ mice. The following were the PCR genotyping primers: WT-primer (5′-ACAGCACGTACATGGAGGTCAGAGGAT-3′), KO primer (5′-TTCTACTCCCTGCATCCATCCACACTT-3′), and WT-R primer (5′-TTTTGAGACAGGACTTTGCCAAGGAGC-3′). Detecting wild-type alleles involved the use of WT and WT-R primers, whereas the detection of mutant alleles involved using KO and WT-R primers. Throughout the experiments, all mice were maintained under specific pathogen-free conditions. Notably, all animal experiments conducted during the study were approved by the Juntendo University Animal Experimental Ethics Committee and adhered to the National Institutes of Health guidelines for animal care.

### 2.4. Preparation of Cell Lysates and Western Blotting

Whole-cell lysates were collected using radio-immunoprecipitation assay buffer at the indicated time points. Sodium dodecyl sulfate–polyacrylamide gel electrophoresis was performed on equal amounts of whole-cell lysates (10–20 mg), followed by protein transfer to polyvinylidene difluoride membranes. Following the blocking step with 5% bovine serum albumin in Tris-buffered saline containing 0.1% Tween-20, membranes were subjected to overnight incubation with the indicated primary antibodies. Anti-TSLP mAbs were used at 1/500 dilution. Anti-JNK, anti-phospho-JNK mAbs, anti-ERK, anti-phospho-ERK, anti-p38MAPK, anti-phospho-p38 MAPK, anti-GAPDH mAbs, and anti-MAP3K19 polyclonal antibodies were used at 1/1000 dilution. The membranes were then subjected to incubation with the appropriate HRP-conjugated secondary antibodies (used at 1/4000 dilution; GE Healthcare, Chicago, IL, USA). Densitometry analysis of Western blot signals, obtained using the ChemiDoc Imaging System (Bio-Rad Laboratories, Hercules, CA, USA) with a linearity of four orders of magnitude, was performed using the image analysis software ImageJ version 1.53.

### 2.5. RNA Interference Assay

The Lipofectamine RNAiMAX transfection agent and oligonucleotides used in this study were obtained from Life Technologies, Inc. (Grand Island, NY, USA) and Invitrogen (Carlsbad, CA, USA). The oligonucleotides were custom-synthesized, and the sense and antisense sequences are detailed in Supplementary Table S1. Following the manufacturer’s guidelines, Lipofectamine RNAiMAX was used to facilitate the transfection of BEAS-2B cells with 20 nM siRNA duplexes. Two distinct sets of siRNA duplexes (siRNA oligo #1 and oligo #2) targeting each protein were utilized to silence human MAP3K19. In addition, the scramble siRNA was included as a negative control. Briefly, the siRNA duplexes and Lipofectamine RNAiMAX were mixed and allowed to incubate at room temperature for 5 min in Opti-MEM I reduced serum medium (Opti-MEM) (Life Technologies, Inc.). Following transfection with the siRNA/Lipofectamine RNAiMAX complexes, the cells were seeded on six-well plates. After a 24 h incubation at 37 °C, the transfected cells were divided into groups and treated for 48 h with the absence (control), TGF-β1 (10 ng/mL), or TWEAK (100 ng/mL). The evaluation of the knockdown efficacy was conducted at the designated time points using quantitative real-time reverse transcription PCR.

### 2.6. Induction of Airway Inflammation

After being anesthetized with isoflurane, the mice were immunized on day 0 via the intraperitoneal (i.p.) injection of 20 μg of ovalbumin (OVA) (Sigma-Aldrich, Burlington, MA, USA) and 2 mg of alum adjuvant (Thermo Fisher Scientific, Yokohama, Japan) (OVA/alum). Following that, on days 7, 8, 9, and 10, intranasal challenges with only 20 μg of OVA were administered to the mice. Negative-control animals were intraperitoneally injected with phosphate-buffered saline (PBS)/alum and challenged with PBS using similar methods.

### 2.7. Characterization of Bronchoalveolar Lavage (BAL) Fluids

A polyethylene tube was used to cannulate the trachea, allowing for the gentle lavage of the lungs with 0.5 mL of PBS containing 10% fetal calf serum (FCS), repeated four times (2 mL of the total BAL fluid). The total cell count was determined using Turk dye exclusion. Cytospins were stained with Diff-Quik (Sysmex International Reagents, Kobe, Japan) to perform the differential cell count. Multiplex bead array assays (Bio-Plex, Bio-Rad Laboratories, Hercules, CA, USA) were used to assay the BAL fluids, following the manufacturer’s instructions. The assay working range, as specified in Supplementary Table S2, spanned from the lower to the upper limit of quantification.

### 2.8. Airway Hyperresponsiveness (AHR) Measurements

Mice underwent anesthesia using pentobarbital and xylazine before being intubated with metal 18-gauge catheters through a tracheostomy. Following intubation, the catheter was immediately connected to the flexiVent™ (SCIREQ, Montreal, QC, Canada). Mechanical ventilation was initiated, maintaining an average breathing frequency of 150 breaths/min. Baseline resistance was measured after saline inhalation, and, subsequently, the mice were exposed to increasing concentrations (0, 6, 12, 24, and 48 mg/mL) of methacholine aerosol. The aerosol was generated using an in-line nebulizer and administered directly through the ventilator for 5 s.

### 2.9. Stimulation of Lung-Draining Lymph Node Cells In Vitro

In an RPMI 1640 medium containing 10% FCS, 10 mM 4-(2-hydroxyethyl)-1-piperazineethanesulfonic acid, 2 mM L-glutamine, 0.1 mg/mL penicillin and streptomycin, and 50 μM 2-mercaptoethanol, lung-draining lymph node (LLN) cells were cultured at a density of 6 × 10^5^ cells/well. The cells were treated with the indicated doses of OVA. The cultures were pulsed with tritiated thymidine ([3H]TdR; 0.5 μCi/well) during the last 6 h of a 48 h culture period to evaluate the proliferative responses. The incorporated radioactivity was measured using a microplate beta counter (PerkinElmer, Winter Street Waltham, MA, USA) after harvesting on a Micro 96 Harvester (Molecular Devices, Sunnyvale, CA, USA).

### 2.10. Histological Analysis of Lung Sections

Mice that did not undergo the bronchial lavage procedure were used for lung harvesting. The lungs were inflated and fixed via the intratracheal instillation of 10% buffered formalin (pH 7.4) under a constant pressure of 25 cm H_2_O for 48 h. Following routine processing that involved successive dehydration, the lungs were embedded in paraffin and sectioned in a coronal plane. Sections with a thickness of 5 μm were stained with hematoxylin and eosin and periodic acid–Schiff to evaluate mucus production. The quantification of the eosinophil infiltration in the lung tissue was conducted anonymously using a five-point scoring system, described as follows: score 0: no eosinophils present; score 1: a few eosinophils observed; score 2: a single-cell-layer ring of eosinophils; score 3: a ring of eosinophils spanning from two to four cell layers; score 4: a ring of eosinophils extending beyond four cell layers. For each lung section, a minimum of 15 different fields were scored, ensuring a robust analysis. The mean scores were derived from six animals. Lung sections were obtained from the same lobe in each mouse, with a minimum of three–four random sections analyzed anonymously.

### 2.11. Immunofluorescence Staining of Lung Sections for TWEAK

For staining lung sections, the following primary antibodies and their respective concentrations were used: goat anti-TWEAK polyclonal antibodies (5 μg/mL), rabbit anti-CD11c monoclonal antibodies (500 ng/mL), and rat anti-CD170 monoclonal antibodies (50 μg/mL). Appropriate secondary antibodies were then applied, including donkey anti-goat IgG conjugated with Alexa Fluor 488 (used at a dilution of 1:250), donkey anti-rabbit IgG conjugated with Alexa Fluor 647 (used at a dilution of 1:200), and donkey anti-rat IgG conjugated with Alexa Fluor 555 (used at a dilution of 1:200). The slides were examined and captured using a Leica TCS SP5 confocal microscope. The resulting images were analyzed with LAS AF software (version 2.6.3, Leica Microsystems CMS GmbH, Wetzlar, Germany) and ImageJ software (NIH, Bethesda, MD, USA). During immunofluorescence staining of lung sections, all fields in each sample were analyzed, and the field with the highest cell concentration was selected to ensure consistency. The number of TWEAK-positive cells was counted in each field, and the mean number per field per section was determined.

### 2.12. Immunohistochemical Staining of Lung Sections

Lung sections were stained with anti-RANTES mAb (used at 1/200 dilution), anti-TSLP polyclonal antibodies (used at 1/800 dilution), anti-E-cadherin mAb (used at 1/50 dilution), and anti-CD3 mAb (used at 1/100 dilution). The sections were incubated with them overnight at 4 °C. They were then incubated with a biotinylated secondary antibody—NICHIREI-Histofine simple-stain MAX-PO (Nichirei, Tokyo, Japan)—for 30 min at room temperature. Slides were incubated with 3,3′-diaminobenzidine tetrahydrochloride and were then counter-stained with hematoxylin.

### 2.13. RNA Isolation and Quantitative RT-PCR

After the last PBS or OVA challenge, lung and trachea tissues were collected from mice and minced. The tissue samples and BEAS-2B bronchial epithelial cells were subjected to RNA isolation using the RNeasy Plus Mini Kit (Qiagen, Valencia, CA, USA) with deoxyribonuclease treatment. Subsequently, cDNA synthesis was performed using the First-Strand complementary DNA (cDNA) Synthesis Kit (GE Healthcare, Little Chalfont, Buckinghamshire, UK). Quantitative real-time reverse transcription PCR (qRT-PCR) was conducted using the Fast SYBR Green Master Mix and an ABI 7500 Fast real-time PCR instrument (Applied Biosystems, Foster City, CA, USA). The qRT-PCR reactions used the gene-specific primer pairs listed in Supplementary Table S3. To analyze the data, the comparative threshold cycle value for glyceraldehyde-3-phosphate dehydrogenase (GAPDH) was used as a normalization factor to account for loading variations in the real-time PCRs.

### 2.14. RANTES Measurements

The concentrations of RANTES protein in culture supernatants collected 48 h after stimulation were measured using human RANTES enzyme-linked immunosorbent assay (ELISA) kits (R&D Systems), following the manufacturer’s instructions.

### 2.15. Statistical Analysis

The normality of the samples was assessed using the D’Agostino–Pearson test. The one-way repeated-measure analysis of variance and Tukey’s multiple-comparisons test, Friedman’s test, and Dunn’s test were employed to evaluate and analyze the differences between multiple groups effectively. Statistical significance was set at *p* ≤ 0.05. All statistical analyses were performed using GraphPad Prism version 8 software (GraphPad Software, San Diego, CA, USA).

## 3. Results

### 3.1. MAP3K19 Suppresses TWEAK Stimulation and RANTES Production

**Figure 1:**
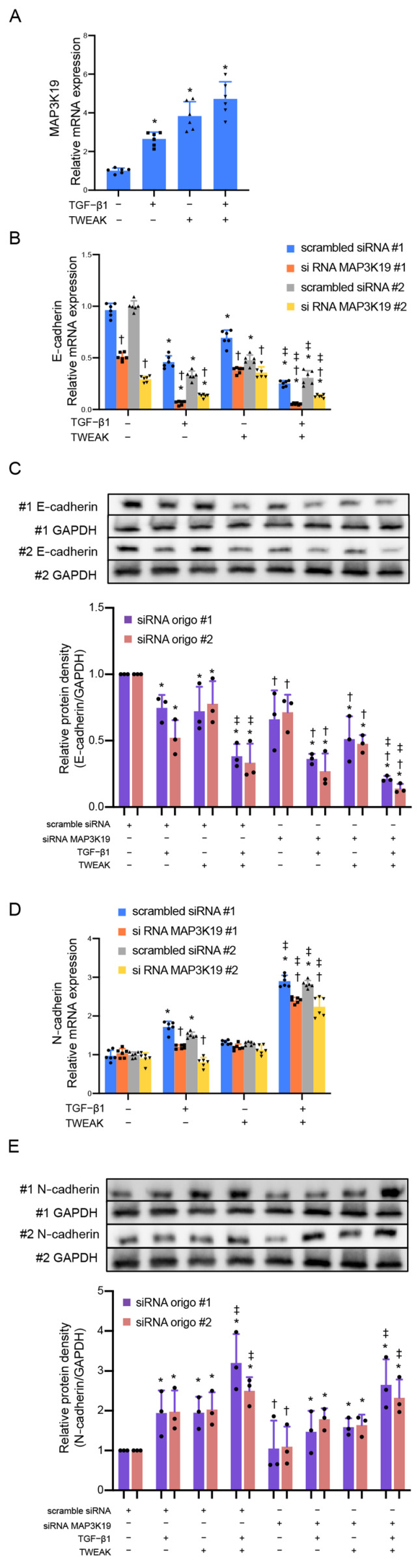
Stimulation with TWEAK and TGF-β1 induced MAP3K19 mRNA expression, and MAP3K19 siRNA enhanced E-cadherin downregulation induced by TWEAK and TGF-β1 in BEAS-2B cells. BEAS-2B cells were treated with TGF-β1 (10 ng/mL), TWEAK (100 ng/mL), or a combination of TWEAK and TGF-β1 for 48 h. The levels of (**A**) MAP3K19, (**B**) E-cadherin, and (**D**) N-cadherin mRNA were analyzed using qRT-PCR. Expression levels were normalized to the housekeeping gene GAPDH and calculated as fold induction compared with the control. Whole-cell lysates were immunoblotted for (**C**) E-cadherin and (**E**) N-cadherin proteins. The membrane was re-probed with an anti-β-actin antibody to confirm equal loading. The density of each band was normalized to β-actin and quantified via densitometry. Data represent the means ± SD of two independent experiments. * *p* < 0.05 compared with each control. ^†^ *p* < 0.05 compared with each scrambled siRNA as control. ^‡^ *p* < 0.05 compared with TGF-β1 alone. Abbreviations: MAP3K19: mitogen-activated protein kinase kinase kinase 19; siRNA: small interfering RNA; mRNA: messenger RNA; TGF: tumor growth factor; SD: standard deviation; qRT-PCR: quantitative reverse transcription polymerase chain reaction; GAPDH: glyceraldehyde-3-phosphate dehydrogenase; TWEAK: tumor necrosis factor-like weak inducer of apoptosis.

We previously demonstrated that the TWEAK receptor Fn14 is expressed on the bronchial epithelial cell line BEAS-2B and that co-stimulation with TWEAK and TGF-β1 induces the EMT [6]. Therefore, we confirmed whether exogenous TWEAK could induce MAP3K19′s expression in BEAS-2B cells. Confluent monolayers of BEAS-2B cells were treated with TWEAK (100 ng/mL) and TGF-β1 (10 ng/mL). Figure 1A shows that TWEAK and TGF-β1 stimulation upregulated the MAP3K19 messenger RNA (mRNA) expression analyzed using quantitative real-time PCR (Figure 1A). Subsequently, siRNA was employed to knock down MAP3K19′s expression, aiming to confirm its involvement (Supplementary Figure S1). We also confirmed that TWEAK or TGF-β1 stimulation alone and co-stimulation with TWEAK and TGF-β1 downregulated the mRNA and E-cadherin protein levels in the BEAS-2B cells and knocked down MAP3K19 using siRNA-enhanced E-cadherin attenuations (Figure 1B,C). However, the knocked-down MAP3K19 attenuated the mRNA expression of N-cadherin, a mesenchymal marker, but not the protein levels, and this mRNA expression effect was upregulated via stimulation with TGF-β1 and co-stimulation with TWEAK and TGF-β1 (Figure 1D,E).

**Figure 2:**
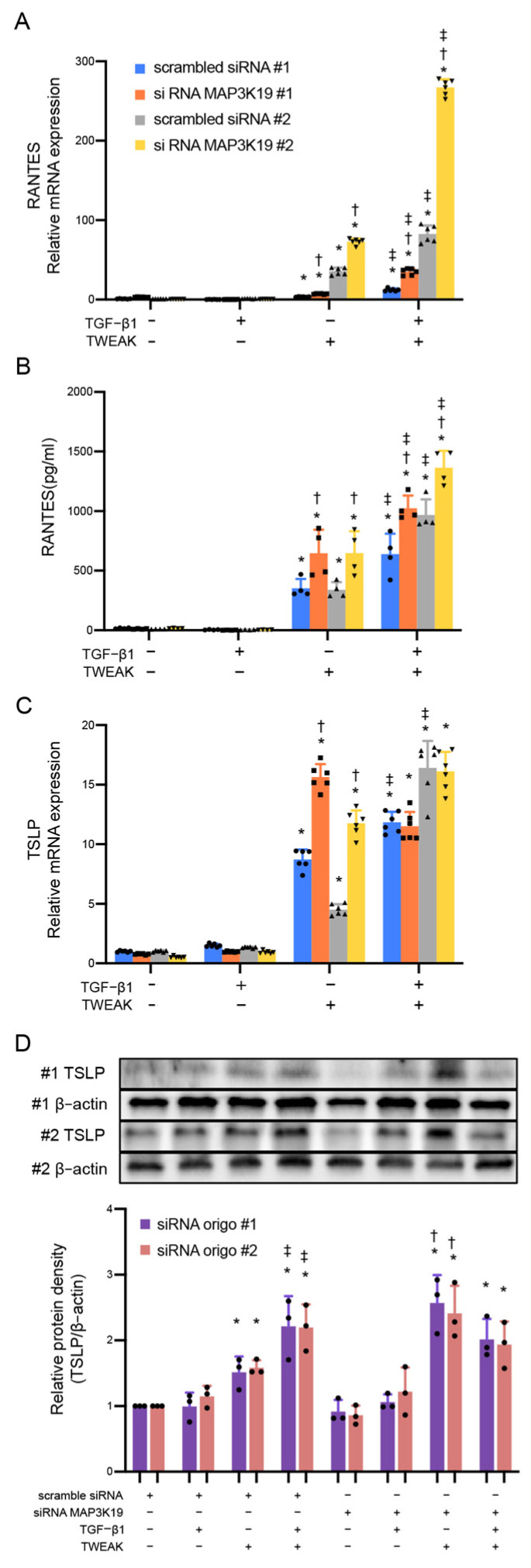
TWEAK induces the production of RANTES in BEAS-2B cells. BEAS-2B cells were treated with TGF-β1 (10 ng/mL), TWEAK (100 ng/mL), or a combination of TWEAK and TGF-β1, as indicated, for 48 h. The levels of (**A**) RANTES and (**C**) TSLP mRNA were analyzed using qRT-PCR. Expression levels were normalized to the housekeeping gene GAPDH and calculated as fold induction compared with the control. (**B**) The levels of RANTES in cell culture supernatants were analyzed using ELISA. (**D**) Whole-cell lysates were immunoblotted for TSLP protein. The membrane was re-probed with an anti-β-actin antibody to confirm equal loading. The density of each band was normalized to β-actin and quantified via densitometry. Data represent the means ± SD of two independent experiments. * *p* < 0.05 compared with each control. ^†^ *p* < 0.05 compared with each scrambled siRNA as control. ^‡^ *p* < 0.05 compared with TWEAK alone. Abbreviations: MAP3K19: mitogen-activated protein kinase kinase kinase 19; siRNA: small interfering RNA; TGF: tumor growth factor; SD: standard deviation; RANTES: regulated upon activation normal T cell express sequence; TWEAK: tumor necrosis factor-like weak inducer of apoptosis; TSLP: thymic stromal lymphoprotein; GAPDH: glyceraldehyde-3-phosphate dehydrogenase; ELISA: enzyme-linked immunosorbent assay.

Our previous reports have shown that co-stimulation with TWEAK and TGF-β1 induces the production of RANTES and TSLP in BEAS-2B cells during the EMT [18]. MAP3K19 knockdown enhanced the mRNA and protein levels of RANTES production in BEAS-2B cells via stimulation with TWEAK and co-stimulation with TWEAK and TGF-β1 (Figure 2A,B). MAP3K19 knockdown also enhanced the mRNA and protein levels of TSLP production via stimulation with TWEAK but not via co-stimulation with TWEAK and TGF-β1 (siRNA oligo #1: *p* = 0.678; oligo #2: *p* = 0.787) (Figure 2C,D).

### 3.2. MAP3K19 Inhibits Phosphorylation of Extracellular Signal-Regulated Kinases (ERKs) by Inducing MAPK Phosphatases-1 (MKP-1)

**Figure 3:**
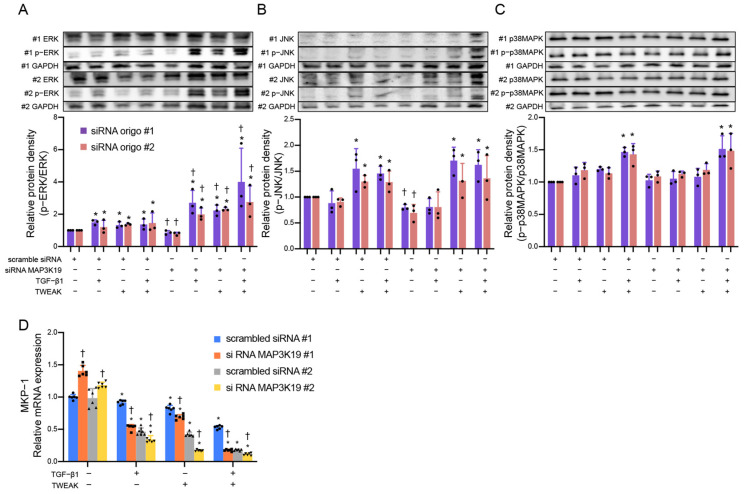
MAP3K19 siRNA enhanced ERK phosphorylation and MKP-1 expression in BEAS-2B cells. BEAS-2B cells were treated with TGF-β1 (10 ng/mL), TWEAK (100 ng/mL), or a combination of TWEAK and TGF-β1 at different times of incubation: ERK, p38 MAPK at 30 min; JNK at 1 h. Whole-cell lysates were immunoblotted for (**A**) ERK, (**B**) JNK, and (**C**) p38 MAPK (upper). The membranes were re-probed with anti-GAPDH antibody to confirm equal loading. The densities of (**A**) ERK, (**B**) JNK, and (**C**) p38 MAPK were normalized with GAPDH and quantified using densitometry (lower). Data represent the means ± SD of three independent experiments. (**D**) BEAS-2B cells were treated with TGF-β1 (10 ng/mL), TWEAK (100 ng/mL), or a combination of TWEAK and TGF-β1, as indicated, for 48 h. MKP-1 mRNA was analyzed using qRT-PCR. Expression levels were normalized to the housekeeping gene GAPDH and calculated as fold induction compared with the control. Data represent the means ± SD of two independent experiments. * *p* < 0.05 compared with each control. ^†^ *p* < 0.05 compared with each scrambled siRNA as control. Abbreviations: MAP3K19: mitogen-activated protein kinase kinase kinase 19; siRNA: small interfering RNA; TGF: tumor growth factor; SD: standard deviation; RANTES: regulated upon activation normal T cell express sequence; TWEAK: tumor necrosis factor-like weak inducer of apoptosis; TSLP: thymic stromal lymphoprotein; ERK: extracellular signal-regulated kinase; GAPDH: glyceraldehyde-3-phosphate dehydrogenase; JNK: Jun N-terminal kinase; MAPK: MAP kinase; qRT-PCR: quantitative reverse transcription polymerase chain reaction.

Previous in vitro studies have shown that MAP3K19 activated the ERK and JNK signaling pathways in vitro kinase assays and that MAP3K19 expression plasmid transiently transfected and activated the ERK and JNK pathways in cancer cell lines [30]. Therefore, we investigated the relationship with the MAPK pathway. Knockdown MAP3K19, using siRNA, showed enhanced ERK phosphorylation (siRNA oligo #1 *p* = 0.038, #2 *p* = 0.042), but not that with JNK (siRNA oligo #1 *p* = 0.443, #2 *p* = 0.813) or p38 MAPK (siRNA oligo #1 *p* = 0.678, #2 *p* = 0.787), suggesting that MAP3K19 suppresses ERK phosphorylation (Figure 3A–C). The MAPK family, including ERK, p38 MAPK, and JNK, is phosphorylated and activated by the MAPK kinase; however, it is dephosphorylated and inactivated by MAPK phosphatases [31]. In addition, we investigated whether MAP3K19 induces the expression of MKP-1, one of the MAPK phosphatases. Figure 3D demonstrates that knockdown MAP3K19 reduces the mRNA expression of MKP-1 (siRNA oligo #1 *p* = 0.014, #2 *p* = 0.039). These findings suggest that MAP3K19 may suppress ERK phosphorylation, and the induction of MKP-1 may be partially involved in this mechanism.

### 3.3. MAP3K19 Suppresses Airway Inflammation in OVA-Induced Asthma Murine Model

**Figure 4:**
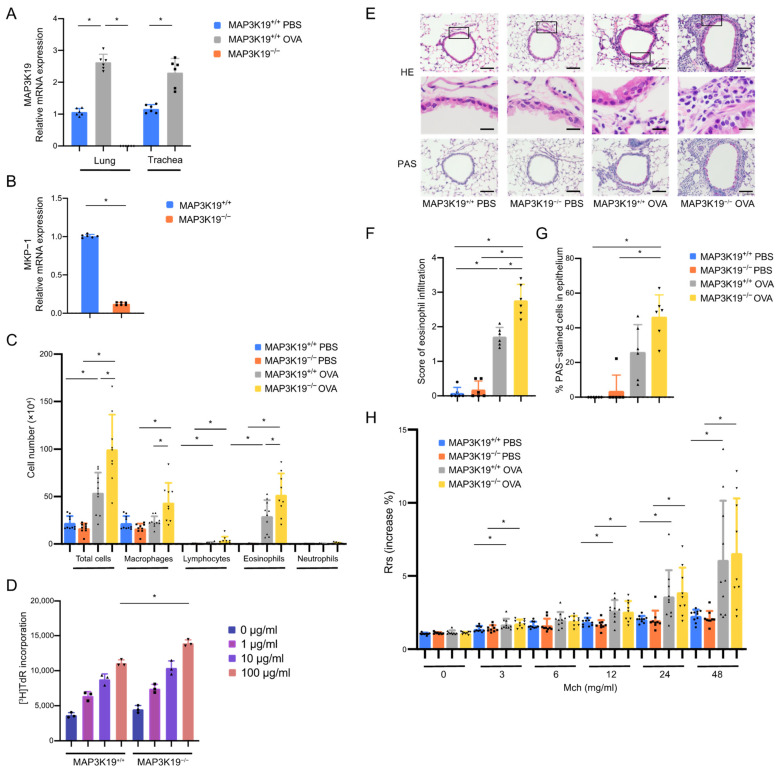
MAP3K19-deficient mice demonstrated exacerbated eosinophilic airway inflammation in the asthma murine model. C57BL/6 mice, which were used as wild-type control mice (MAP3K19^+/+^ mice), and mice with absent MAP3K19 (MAP3K19^−/−^ mice) were immunized with OVA and subsequently challenged intranasally with OVA. (**A**,**B**) Lung tissues from MAP3K19^+/+^ and MAP3K19^−/−^ mice were removed and minced. (**A**) The lung and trachea tissues from OVA-sensitized/challenged MAP3K19^+/+^ mice (MAP3K19^+/+^ OVA) and control PBS-sensitized/challenged MAP3K19^+/+^ mice (MAP3K19^+/+^ PBS) were removed and minced 24 h after the last challenge. The (**A**) MAP3K19 mRNA expression levels in lung and trachea tissues and (**B**) MKP-1 mRNA expression levels in lung tissues were analyzed using qRT-PCRs. Expression levels were normalized to the housekeeping gene GAPDH and calculated as fold induction compared with the control (n = four–five mice per group). (**C**) BAL fluids were collected 24 h after the last injection. The cellular compositions of airway infiltrates are shown (n = 9–11 mice per group). (**D**) Lung-draining lymph node cells were isolated and cultured with indicated concentrations of OVA. For estimating proliferation, 0.5 µCi of thymidine ([^3^H]TdR) was added during the last 6 h of a 72 h culture. (**E**) Lung tissue was stained with H&E and PAS. Scale bar: 100 μm (H&E Upper and PAS), 20 μm (H&E Lower) (n = six mice per group). The tissues within the box shown at the H&E Upper panel is enlarged to the H&E Lower panel. (**F**) The eosinophilic infiltration is defined as the average of the scores. (**G**) Airway mucus levels are expressed as the percentage of PAS-positive cells to total epithelial cells. (**H**) On the day following the last injection, individual mice were assessed for AHR. Results are presented as the mean respiratory system resistance (Rrs) ± SD in each group after exposure to increasing concentrations of inhaled methacholine (n = 9–10 mice per group). Data represent the means ± SD. * *p* < 0.05. Abbreviations: MAP3K19: mitogen-activated protein kinase kinase kinase 19; AHR: airway hyperresponsiveness; OVA: ovalbumin; SD: standard deviation; PBS: phosphate-buffered saline; MKP-1: MAPK phosphatases-1; qRT-PCR: quantitative reverse transcription polymerase chain reaction; GADPH: glyceraldehyde-3-phosphate dehydrogenase; BAL: bronchoalveolar lavage; H&E: hematoxylin and eosin; PAS: periodic acid–Schiff.

Our in vitro studies suggested that MAP3K19 is involved in type 2 inflammation and cellular EMT processes that may contribute to airway inflammation in patients with asthma; therefore, we investigated whether MAP3K19 knockout is involved in airway inflammation in a murine model of asthma. We first confirmed that the expression of MAP3K19 mRNA was upregulated in the lungs (*p* = 0.003) and tracheas (*p* = 0.002) of OVA-sensitized/challenged female C57BL/6 mice, used as wild-type control mice (MAP3K19^+/+^OVA) (Figure 4A). MAP3K19 mRNA could not be detected in the lungs of the OVA-sensitized/challenged MAP3K19^−/−^ mice (MAP3K19^−/−^OVA) (Figure 4A). The MKP-1 mRNA expression in the lungs of the MAP3K19^−/−^ mice was decreased compared with that of the wild-type control mice (Figure 4B, *p* = 0.002). The MAP3K19^−/−^OVA mice had a significantly higher total cell number (*p* < 0.001) and significantly more eosinophils (*p* = 0.007) in the BAL fluids compared with the control MAP3K19^+/+^OVA mice (Figure 4C). The OVA-specific proliferative response was significantly increased in the lymph node cells of the MAP3K19^−/−^OVA mice compared with the MAP3K19^+/+^OVA mice (Figure 4D, *p* = 0.030). Histological analysis of the lung sections showed that the eosinophilic infiltration was significantly increased in the MAP3K19^−/−^OVA mice compared with the MAP3K19^+/+^OVA mice (Figure 4E,F, *p* = 0.033). The mucus overproduction tended to increase; however, there were no significant differences in the mucus overproduction or the development of AHR between the MAP3K19^−/−^OVA and MAP3K19^+/+^OVA mice (Figure 4G, *p* = 0.103, and Figure 4H, *p* > 0.999).

### 3.4. MAP3K19 Suppresses RANTES Production in OVA-Induced Asthma Murine Model

**Figure 5:**
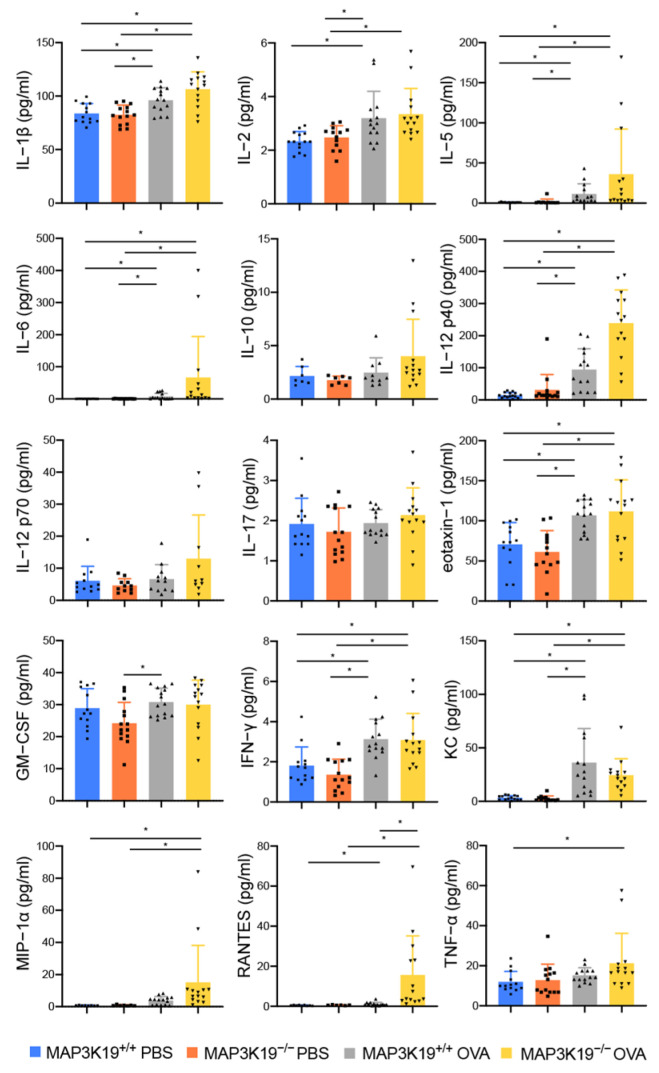
Comparison of secreted cytokines in BAL fluids from OVA-sensitized/challenged MAP3K19-deficient mice (MAP3K19^−/−^ mice) and control mice (MAP3K19^+/+^ mice). BAL fluids were collected 24 h after the last injection. The concentrations of IL-1β, IL-2, IL-5, IL-6, IL-10, IL-12p40, IL-12p70, IL-17, eotaxin-1, GM-CSF, IFN-γ, keratinocyte-derived cytokine (KC), MIP-1α, RANTES, and TNF-α in the BAL fluids were measured using multiplex bead array assays. Data represent the means ± SD (n = 11–15 mice per group). * *p* < 0.05. Abbreviations: BAL: bronchoalveolar lavage; MAP3K19: mitogen-activated protein kinase kinase kinase 19; SD: standard deviation; RANTES: regulated upon activation normal T cell express sequence; TNF: tumor necrosis factor; IL: interleukin; INF: interferon; GM-CSF: granulocyte-macrophage colony-stimulating factor.

We previously demonstrated that TWEAK promotes NF-κB activation and TGF-β1-induced TSLP and RANTES production in bronchial epithelial cells [17,18]. Consequently, we investigated whether TWEAK, TSLP, and RANTES are involved in exacerbating airway inflammation with MAP3K19 knockout. The RANTES in the BAL fluids was significantly increased in the MAP3K19^−/−^OVA mice compared with the MAP3K19^+/+^OVA mice (Figure 5).

**Figure 6:**
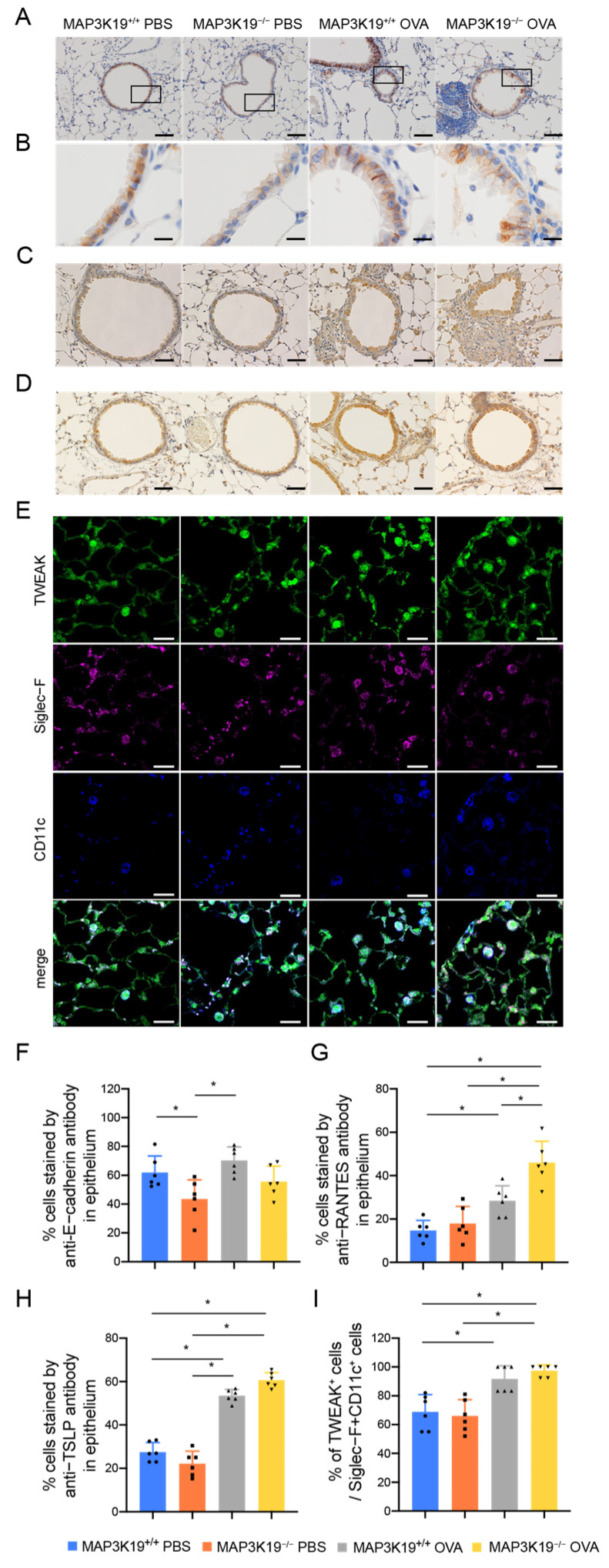
MAP3K19-deficient mice enhanced RANTES production in an OVA-induced asthma murine model. Lung tissues from mice were removed and stained with (**A**,**B**,**F**) E-cadherin, (**C**,**G**) RANTES, (**D**,**H**) TSLP, and (**E**,**I**) TWEAK. The (**A**) panel displays tissues within the box, which are enlarged in the (**B**) panel. (**E**) Lung tissue samples were stained with immunofluorescent antibodies and demonstrated single-color and merged-color display with TWEAK (green), Siglec-F (magenta), and CD11c (blue). Scale bars: (**B**,**E**) 20 μm, (**C**,**D**) 100 μm. Graph showing the percentage of (**F**) E-cadherin-, (**G**) RANTES-, and (**H**) TSLP-positive cells in bronchial epithelial cells in each lung section and (**I**) the percentage of TWEAK-positive cells per CD11c- and Siglec-F-positive alveolar macrophages in each lung section. Data represent the means ± SD (n = 6 mice per group). * *p* < 0.05. Abbreviations: MAP3K19: mitogen-activated protein kinase kinase kinase 19; SD: standard deviation; RANTES: regulated upon activation normal T cell express sequence; TWEAK: tumor necrosis factor-like weak inducer of apoptosis; TSLP: thymic stromal lymphoprotein.

As MAP3K19 knockdown enhanced the reduction in E-cadherin mRNA in the BEAS-2B cells, we analyzed the E-cadherin expression in mouse bronchial epithelia. We found that E-cadherin was reduced in the bronchial epithelia of the MAP3K19 knockout mice (Figure 6A,B,F). However, no further reduction in E-cadherin expression was observed in the OVA-sensitized/challenged mice. Histological analysis of lung sections also showed that RANTES-positive cells in the bronchial epithelium were significantly increased in the MAP3K19^−/−^OVA mice compared with the MAP3K19^+/+^OVA mice (Figure 6C,G). TSLP was significantly expressed in the epithelial cells of the OVA-induced asthma murine model; however, there was no difference in the number of TSLP-positive cells between the MAP3K19^−/−^OVA and MAP3K19^+/+^OVA mice (Figure 6D,H). TWEAK was significantly expressed in infiltrating cells in alveolar spaces, mostly CD11c- and Siglec-F-positive alveolar macrophages, of the MAP3K19^−/−^OVA mice, compared with that in the MAP3K19^+/+^OVA control mice; however, there was no difference in the number of TWEAK-expressing cells between the MAP3K19^−/−^OVA and MAP3K19^+/+^OVA mice (Figure 6E,I). In addition, there was no difference in the number of CD3-positive T cells among these mice (Supplementary Figure S2). These findings suggested that the MAP3K19 knockout enhanced the RANTES production in the bronchial epithelial cells.

## 4. Discussion

To the best of our knowledge, this is the first study to demonstrate the following findings: (i) TWEAK and TGF-β1 stimulation upregulated the MAP3K19 mRNA expression, and MAP3K19 knockdown enhanced the E-cadherin reduction and RANTES production, caused by stimulation with TWEAK alone and co-stimulation with TGF-β1 and TWEAK in cultured bronchial epithelial cells; (ii) MAP3K19 may suppress ERK phosphorylation and induce MKP-1 expression; and (iii) the expression of MAP3K19 mRNA was upregulated in the lungs and tracheas of mice in the OVA-induced asthma murine model, and the MAP3K19 knockout mice had worsened eosinophilic airway inflammation and increased production of RANTES in the airway epithelium compared with the wild-type mice.

The loss of MAP3K19 enhanced the TWEAK-induced progressive adhesion factor attenuation and the production of cytokine and chemokines associated with type 2 airway inflammation, including RANTES and TSLP, and also enhanced co-stimulation (TWEAK and TGF-β1)-induced progressive adhesion factor attenuation and RANTES production. These findings suggest that MAP3K19 might be involved in the phenomenon in airway epithelia when some external stimuli induce the disruption of tight junctions and the production of epithelial cytokines. Previous in vitro culture systems have shown that MAP3K19 siRNA or MAP3K19 inhibitors reduced the TGF-β-induced phospho-Smad2/3 nuclear translocation in an A549 human lung adenocarcinoma cell line, THP-1 human monocytes/macrophages, Human Embryonic Kidney cells, and HeLa cervical cancer cells [3,4]. In the present study, the MAP3K19 siRNA in the bronchial epithelial cells reduced the TGF-β1-induced N-cadherin and MKP-1 mRNA expression and induced ERK phosphorylation, consistent with previous reports of the TGF-β-induced reduction in Smad2/3 phosphorylation in HeLa cells [3]. Our previous study showed that epithelial adhesion factor attenuation and RANTES production induced via TWEAK stimulation or a combination of TWEAK and TGF-β1 were involved through non-Smad signaling pathways, including the NF-κB and MAPK pathways [18]. Combined with the previous findings stating that TGF-β-induced activated ERK can phosphorylate smad2/3 and increase the duration of transcriptional activity in normal mesenchymal cells [32,33], suggested that cross talk exists between the ERK and Smad signaling in EMT cells. Conversely, Hoang VT. et al. have reported that MAP3K19 expression plasmid transiently transfected and activated MAPK family members, including ERK and JNK, in HEK293 cells [30]. Moreover, an in vitro kinase assay has shown that MAP3K19 directly phosphorylates MAP2 kinases [30]. MAP3K19 knockout attenuated the E-cadherin expression in cultured bronchial epithelial cells and mouse bronchial epithelia, whereas MAP3K19 siRNA had only a partial suppressive effect on the N-cadherin expression, induced via co-stimulation with TWEAK and TGF-β1 in bronchial epithelial cells, and the E-cadherin expression was not decreased in OVA-sensitized/challenged mice. These results suggest that MAP3K19 is involved in maintaining airway epithelial tight junctions but may be partially involved in the EMT. A difference was noted in the target cell lines in our findings and those of the previous reports; however, further investigations are needed to address these discrepancies.

Contrary to previous reports that mice treated with a small-molecule MAP3K19 inhibitor demonstrated significantly suppressed bleomycin-induced pulmonary fibrosis and cigarette smoke-induced inflammation and emphysema [3,4,5], our findings suggest that MAP3K19 knockout resulted in progressive OVA-induced allergic airway inflammation, without increased AHR, in the murine model. The reason for the discrepancy between the findings on the MAP3K19 inhibitor in the murine models of fibrosis and emphysema and that of our findings in the asthma murine model using the MAP3K19 knockout mouse is unknown; however, there are differences between the asthma pathology focused on bronchial epithelial cells and those of the previous reports focused on macrophages and fibroblasts. Consequently, the MAP3K19 kinase function remains unclear, and our findings indicating that MAP3K19 inhibition exacerbates asthma will shed further light on this topic.

In our cell experiments, TWEAK enhanced the RANTES and TSLP production in the loss of MAP3K19. However, co-stimulation with TGF-β1 and TWEAK in the MAP3K19 knockdown cultured cells affected the RANTES production but not the TSLP production. Similarly, in our mouse experiments, we observed increased RANTES production in an asthma model of MAP3K19^−/−^ mice, whereas the increased TSLP- and TWEAK-expressing cells in the MAP3K19^−/−^ mice were no different from those of the wild type. These findings suggested that MAP3K19 is not involved in TWEAK and TSLP production in the OVA-induced asthma model because MAP3K19 is downstream of TWEAK. Additional analyses are required to investigate the mechanistic differences in the production of TSLP and RANTES, especially concerning promoter regions.

Our study has certain limitations. First, the MAP3K19 kinase function could not be clearly determined. The second limitation is the use of BEAS-2B cell monolayers, which show characteristics of non-differentiated basal human bronchial epithelial cells. Third, we could not determine the exact concentration of TWEAK to be used in the present study. There is no evidence for the exact physiological concentration for using TWEAK in experiments on lung diseases, including asthma; however, stimulation with 100 ng/mL of TWEAK was performed in the present study, as reported previously [17,34,35,36,37]. Fourth, we could not determine the optimal protocol of the OVA-induced asthma murine model to clarify the involvement of the EMT and the worsened eosinophilic airway inflammation effects of the MAP3K19 knockout mice. There are many protocols for OVA-induced asthma murine models, and the OVA doses in the sensitization and challenge phases are varied [38,39]. There is no clear rationale for the dose of OVA; however, 20 μg of OVA was used in the sensitization and challenge phases in this study, as previously reported [40,41,42,43,44,45,46]. Finally, we could not sufficiently analyze the asthma model using MAP3K19 knockout mice, and further studies are required to determine how non-Smad signaling pathways, such as the NF-κB and MAPK pathways and MKP-1, are involved in these knockout mice. However, studies on the cell culture model and asthma murine model have suggested that MAP3K19 is involved in TWEAK, TGF-β1, and their combination, which induced E-cadherin reduction, the production of RANTES, ERK phosphorylation, MKP-1 expression, and the OVA-induced asthma model pathology, including RANTES production.

In conclusion, we revealed that TWEAK and TGF-β1 stimulation and an OVA-induced asthma murine model upregulates MAP3K19 mRNA expression; MAP3K19 suppresses TWEAK-stimulated airway epithelial responses, including adhesion molecule attenuation and RANTES production; and it suppresses allergic airway inflammation in a mouse model of asthma, suggesting that MAP3K19 regulates allergic airway inflammation in patients with asthma.

## Data Availability

Data are contained within the article and Supplementary Materials.

## Supplementary Materials

The supporting information can be downloaded at https://www.mdpi.com/article/10.3390/cimb45110559/s1.

## Abbreviations

COPD: chronic obstructive pulmonary disease; DAB: 3,3′-diaminobenzidine tetrahydrochloride; EMT: epithelial–mesenchymal transition; ERK: extracellular signal-regulated kinase; Fn14: fibroblast growth factor-inducible 14; GAPDH: glyceraldehyde-3-phosphate dehydrogenase; IL: interleukin; JNK: Jun N-terminal kinase; MAP: mitogen-activated protein; MAPK: MAP kinase; MAP3K19: MAP kinase kinase kinase 19; MKP-1: MAPK phosphatases-1; NF-κB: nuclear factor κB; RANTES: regulated upon activation normal T cell express sequence; TARC: thymus and activation-regulated chemokine; TGF: transforming growth factor; Th2: type 2 T helper cells; TNF: tumor necrosis factor; TSLP: thymic stromal lymphopoietin; TWEAK: TNF-like weak inducer of apoptosis.

## References

[1] Arthur J.S., Ley S.C. (2013). Mitogen-activated protein kinases in innate immunity. Nat. Rev. Immunol..

[2] Meister M., Tomasovic A., Banning A., Tikkanen R. (2013). Mitogen-Activated Protein (MAP) Kinase Scaffolding Proteins: A Recount. Int. J. Mol. Sci..

[3] Boehme S.A., Franz-Bacon K., DiTirro D.N., Ly T.W., Bacon K.B. (2016). MAP3K19 Is a Novel Regulator of TGF-beta Signaling That Impacts Bleomycin-Induced Lung Injury and Pulmonary Fibrosis. PLoS ONE.

[4] Boehme S.A., Franz-Bacon K., Ludka J., DiTirro D.N., Ly T.W., Bacon K.B. (2016). MAP3K19 Is Overexpressed in COPD and Is a Central Mediator of Cigarette Smoke-Induced Pulmonary Inflammation and Lower Airway Destruction. PLoS ONE.

[5] Jones I.C., Espindola M.S., Narayanan R., Coelho A.L., Habiel D.M., Boehme S.A., Ly T.W., Bacon K.B., Hogaboam C.M. (2019). Targeting MAP3K19 prevents human lung myofibroblast activation both in vitro and in a humanized SCID model of idiopathic pulmonary fibrosis. Sci. Rep..

[6] Itoigawa Y., Harada N., Harada S., Katsura Y., Makino F., Ito J., Nurwidya F., Kato M., Takahashi F., Atsuta R. (2015). TWEAK enhances TGF-beta-induced epithelial-mesenchymal transition in human bronchial epithelial cells. Respir. Res..

[7] Nguyen K., Tran M.N., Rivera A., Cheng T., Windsor G.O., Chabot A.B., Cavanaugh J.E., Collins-Burow B.M., Lee S.B., Drewry D.H. (2022). MAP3K Family Review and Correlations with Patient Survival Outcomes in Various Cancer Types. Front. Biosci..

[8] Ijaz T., Pazdrak K., Kalita M., Konig R., Choudhary S., Tian B., Boldogh I., Brasier A.R. (2014). Systems biology approaches to understanding Epithelial Mesenchymal Transition (EMT) in mucosal remodeling and signaling in asthma. World Allergy Organ. J..

[9] Lambrecht B.N., Hammad H. (2012). The airway epithelium in asthma. Nat. Med..

[10] Johnson J.R., Roos A., Berg T., Nord M., Fuxe J. (2011). Chronic respiratory aeroallergen exposure in mice induces epithelial-mesenchymal transition in the large airways. PLoS ONE.

[11] Yasukawa A., Hosoki K., Toda M., Miyake Y., Matsushima Y., Matsumoto T., Boveda-Ruiz D., Gil-Bernabe P., Nagao M., Sugimoto M. (2013). Eosinophils promote epithelial to mesenchymal transition of bronchial epithelial cells. PLoS ONE.

[12] Thiery J.P. (2002). Epithelial-mesenchymal transitions in tumour progression. Nat. Rev. Cancer.

[13] Kalluri R., Weinberg R.A. (2009). The basics of epithelial-mesenchymal transition. J. Clin. Investig..

[14] Thiery J.P., Acloque H., Huang R.Y., Nieto M.A. (2009). Epithelial-mesenchymal transitions in development and disease. Cell.

[15] Puddicombe S.M., Polosa R., Richter A., Krishna M.T., Howarth P.H., Holgate S.T., Davies D.E. (2000). Involvement of the epidermal growth factor receptor in epithelial repair in asthma. FASEB J..

[16] Burkly L.C., Michaelson J.S., Zheng T.S. (2011). TWEAK/Fn14 pathway: An immunological switch for shaping tissue responses. Immunol. Rev..

[17] Harada N., Nakayama M., Nakano H., Fukuchi Y., Yagita H., Okumura K. (2002). Pro-inflammatory effect of TWEAK/Fn14 interaction on human umbilical vein endothelial cells. Biochem. Biophys. Res. Commun..

[18] Matsuno K., Harada N., Harada S., Takeshige T., Ishimori A., Itoigawa Y., Katsura Y., Kodama Y., Makino F., Ito H. (2018). Combination of TWEAK and TGF-beta1 induces the production of TSLP, RANTES, and TARC in BEAS-2B human bronchial epithelial cells during epithelial-mesenchymal transition. Exp. Lung Res..

[19] Zhu C., Zhang L., Liu Z., Li C., Bai Y. (2018). TWEAK/Fn14 interaction induces proliferation and migration in human airway smooth muscle cells via activating the NF-kappaB pathway. J. Cell Biochem..

[20] Kamijo S., Nakajima A., Kamata K., Kurosawa H., Yagita H., Okumura K. (2008). Involvement of TWEAK/Fn14 interaction in the synovial inflammation of RA. Rheumatology.

[21] Schwartz N., Su L., Burkly L.C., Mackay M., Aranow C., Kollaros M., Michaelson J.S., Rovin B., Putterman C. (2006). Urinary TWEAK and the activity of lupus nephritis. J. Autoimmun..

[22] Serafini B., Magliozzi R., Rosicarelli B., Reynolds R., Zheng T.S., Aloisi F. (2008). Expression of TWEAK and its receptor Fn14 in the multiple sclerosis brain: Implications for inflammatory tissue injury. J. Neuropathol. Exp. Neurol..

[23] Winkles J.A. (2008). The TWEAK-Fn14 cytokine-receptor axis: Discovery, biology and therapeutic targeting. Nat. Rev. Drug Discov..

[24] Soumelis V., Reche P.A., Kanzler H., Yuan W., Edward G., Homey B., Gilliet M., Ho S., Antonenko S., Lauerma A. (2002). Human epithelial cells trigger dendritic cell mediated allergic inflammation by producing TSLP. Nat. Immunol..

[25] Ziegler S.F., Artis D. (2010). Sensing the outside world: TSLP regulates barrier immunity. Nat. Immunol..

[26] Carr T.F., Berdnikovs S., Simon H.U., Bochner B.S., Rosenwasser L.J. (2016). Eosinophilic bioactivities in severe asthma. World Allergy Organ. J..

[27] Chvatchko Y., Proudfoot A.E.I., Buser R., Juillard P., Alouani S., Kosco-Vilbois M., Coyle A.J., Nibbs R.J., Graham G., Offord R.E. (2003). Inhibition of airway inflammation by amino-terminally modified RANTES/CC chemokine ligand 5 analogues is not mediated through CCR3. J. Immunol..

[28] Kim S.Y., Kim J.D., Sol I.S., Kim M.J., Na Kim M., Hong J.Y., Kim H.R., Kim Y.H., Lee Y.J., Kim K.W. (2018). Sputum TWEAK expression correlates with severity and degree of control in non-eosinophilic childhood asthma. Pediatr. Allergy Immunol..

[29] Cuarental L., Sucunza-Saenz D., Valino-Rivas L., Fernandez-Fernandez B., Sanz A.B., Ortiz A., José Vaquero J., Sanchez-Niño M.D. (2019). MAP3K kinases and kidney injury. Nefrologia.

[30] Hoang V.T., Nyswaner K., Torres-Ayuso P., Brognard J. (2020). The protein kinase MAP3K19 phosphorylates MAP2Ks and thereby activates ERK and JNK kinases and increases viability of KRAS-mutant lung cancer cells. J. Biol. Chem..

[31] Hoppstadter J., Ammit A.J. (2019). Role of Dual-Specificity Phosphatase 1 in Glucocorticoid-Driven Anti-inflammatory Responses. Front. Immunol..

[32] Hough C., Radu M., Dore J.J. (2012). Tgf-beta induced Erk phosphorylation of smad linker region regulates smad signaling. PLoS ONE.

[33] Hayashida T., Decaestecker M., Schnaper H.W. (2003). Cross-talk between ERK MAP kinase and Smad signaling pathways enhances TGF-beta-dependent responses in human mesangial cells. FASEB J..

[34] Chen T., Guo Z.P., Li M.M., Li J.Y., Jiao X.Y., Zhang Y.H., Liu H.J. (2011). Tumour necrosis factor-like weak inducer of apoptosis (TWEAK), an important mediator of endothelial inflammation, is associated with the pathogenesis of Henoch-Schonlein purpura. Clin. Exp. Immunol..

[35] Tran N.L., McDonough W.S., Savitch B.A., Sawyer T.F., Winkles J.A., Berens M.E. (2005). The tumor necrosis factor-like weak inducer of apoptosis (TWEAK)-fibroblast growth factor-inducible 14 (Fn14) signaling system regulates glioma cell survival via NFkappaB pathway activation and BCL-XL/BCL-W expression. J. Biol. Chem..

[36] Vendrell J., Maymo-Masip E., Tinahones F., Garcia-Espana A., Megia A., Caubet E., García-Fuentes E., Chacón M.R. (2010). Tumor necrosis-like weak inducer of apoptosis as a proinflammatory cytokine in human adipocyte cells: Up-regulation in severe obesity is mediated by inflammation but not hypoxia. J. Clin. Endocrinol. Metab..

[37] Xu H., Okamoto A., Ichikawa J., Ando T., Tasaka K., Masuyama K., Ogawa H., Yagita H., Okumura K., Nakao A. (2004). TWEAK/Fn14 interaction stimulates human bronchial epithelial cells to produce IL-8 and GM-CSF. Biochem. Biophys. Res. Commun..

[38] Azman S., Sekar M., Bonam S.R., Gan S.H., Wahidin S., Lum P.T., Dhadde S.B. (2021). Traditional Medicinal Plants Conferring Protection Against Ovalbumin-Induced Asthma in Experimental Animals: A Review. J. Asthma Allergy.

[39] Kianmeher M., Ghorani V., Boskabady M.H. (2016). Animal Model of Asthma, Various Methods and Measured Parameters: A Methodological Review. Iran. J. Allergy Asthma Immunol..

[40] Behrendt A.K., Hansen G. (2010). CD27 costimulation is not critical for the development of asthma and respiratory tolerance in a murine model. Immunol. Lett..

[41] Makino F., Ito J., Abe Y., Harada N., Kamachi F., Yagita H., Takahashi K., Okumura K., Akiba H. (2012). Blockade of CD70-CD27 interaction inhibits induction of allergic lung inflammation in mice. Am. J. Respir. Cell Mol. Biol..

[42] Behrendt A.K., Meyer-Bahlburg A., Hansen G. (2012). CD137 deficiency does not affect development of airway inflammation or respiratory tolerance induction in murine models. Clin. Exp. Immunol..

[43] Busse M., Krech M., Meyer-Bahlburg A., Hennig C., Hansen G. (2012). ICOS mediates the generation and function of CD4+CD25+Foxp3+ regulatory T cells conveying respiratory tolerance. J. Immunol..

[44] Jirmo A.C., Daluege K., Happle C., Albrecht M., Dittrich A.M., Busse M., Habener A., Skuljec J., Hansen G. (2016). IL-27 Is Essential for Suppression of Experimental Allergic Asthma by the TLR7/8 Agonist R848 (Resiquimod). J. Immunol..

[45] Happle C., Jirmo A.C., Meyer-Bahlburg A., Habener A., Hoymann H.G., Hennig C., Skuljec J., Hansen G. (2018). B cells control maternofetal priming of allergy and tolerance in a murine model of allergic airway inflammation. J. Allergy Clin. Immunol..

[46] Jirmo A.C., Busse M., Happle C., Skuljec J., Dalüge K., Habener A., Grychtol R., DeLuca D.S., Breiholz O.D., Prinz I. (2020). IL-17 regulates DC migration to the peribronchial LNs and allergen presentation in experimental allergic asthma. Eur. J. Immunol..

