# Supplementary material for: MAP3K19 Affects TWEAK-Induced Response in Cultured Bronchial Epithelial Cells and Regulates Allergic Airway Inflammation in an Asthma Murine Model
Source: Curr Issues Mol Biol. 2023 Nov 8;45(11):8907–24. doi: 10.3390/cimb45110559 (PMC10670632; doi:10.3390/cimb45110559)
Supplement: Supplementary file 1 [file cimb-45-00559-s001.zip › CIMB-2433578 R1 Supplementary Figure S1 231107.pdf]

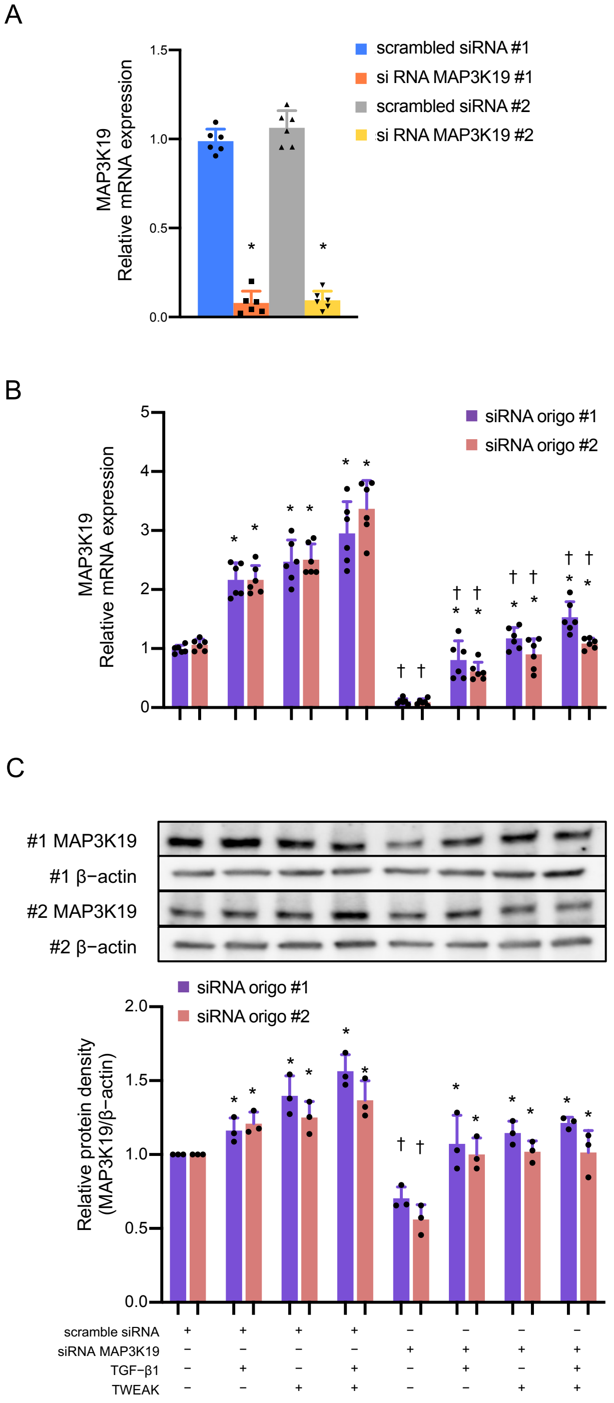

Supplementary Figure S1. MAP3K19 siRNA down-regulated the mRNA and protein levels of MA.P3K19 in BEAS-2B cells. BEAS-2B cells were treated with TGF-βl (10 ng/ml), TWEAK (100 ng/ml), or combination with TWEAK and TGF-βl for 48 h. (A, B) The leves of MAP3Kl9 mRNA were analyzed by qRT-PCR. Expression levels were normalized to the housekeeping gene GAPDH and calculated as fold induction in comparison to the control. (C) Whole cell lysates were immunoblotted for MAP3K19 protein. The membrane was re-probed with anti-β-actin antibody to confirm equal loading. The density of each band was normalized to β-actin and quantified by densitometry. Data represent the means ± SD of two independent experiments. **p* < 0.05 compared with each control. †*p* < 0.05 compared with each scrambled siRNA as control. Abbreviations: MAP3Kl9: Mitogen-Activated Protein Kinase Kinase Kinase 19; siRNA: small interfering RNA; TGF: tumor growth factor; SD: standard deviation; TWEAK: tumor necrosis factor-like weak inducer of apoptosis.
