# Supplementary material for: MAP3K19 Affects TWEAK-Induced Response in Cultured Bronchial Epithelial Cells and Regulates Allergic Airway Inflammation in an Asthma Murine Model
Source: Curr Issues Mol Biol. 2023 Nov 8;45(11):8907–24. doi: 10.3390/cimb45110559 (PMC10670632; doi:10.3390/cimb45110559)
Supplement: Supplementary file 1 [file cimb-45-00559-s001.zip › CIMB-2433578 R1 Supplementary Figure S2 231107.pdf]

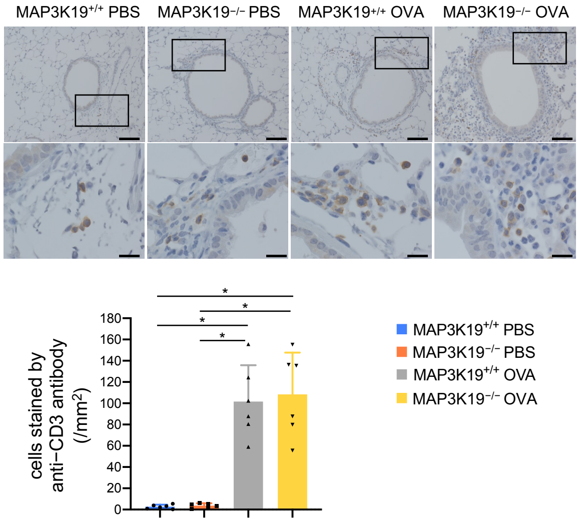

Supplementary Figure S2. There is no significant difference in the number of CD3-positive T cells between WT and MAP3K19-deficient. Lung tissues from mice were removed and were stained with anti-CD3 antibody. Scale bars: 20 µm (upper panels), 100 µm (lower panels). Graph showing the percentage of CD3-positive T cells in each lung section. Data represent the means ± SD (n = 6 mice per group). **p* < 0.05. Abbreviations: MAP3K19: Mitogen-Activated Protein Kinase Kinase Kinase 19; SD: standard deviation.
